# Synergistic antitumor effects of polysaccharides and anthocyanins from *Lycium ruthenicum* Murr. on human colorectal carcinoma LoVo cells and the molecular mechanism

**DOI:** 10.1002/fsn3.2892

**Published:** 2022-04-18

**Authors:** Xinshu Qin, Xingyu Wang, Ke Xu, Xingbin Yang, Qing Wang, Chao Liu, Xinkun Wang, Xu Guo, Jinyue Sun, Lin Li, Shiqi Li

**Affiliations:** ^1^ Key Laboratory of Novel Food Resources Processing Ministry of Agriculture and Rural Affairs Key Laboratory of Agro‐Products Processing Technology of Shandong Province Institute of Agro‐Food Science and Technology Shandong Academy of Agricultural Sciences Ji'nan China; ^2^ Shaanxi Engineering Laboratory for Food Green Processing and Safety Control Shaanxi Key Laboratory for Hazard Factors Assessment in Processing and Storage of Agricultural Products College of Food Engineering and Nutritional Science Shaanxi Normal University Xi'an China; ^3^ Department of Joint Surgery, Hong Hui Hospital Xi'an Jiaotong University Xi'an China; ^4^ Santa Barbara City College University of California Santa Barbara Santa Barbara California USA; ^5^ Department of Material Science and Engineering Queen Mary University of London Engineering School Northwestern Polytechnical University Xi'an China

**Keywords:** anthocyanins, antitumor activities, *Lycium ruthenicum* Murr., polysaccharides, synergistic effect

## Abstract

The antitumor effects of *Lycium ruthenicum* Murr. polysaccharides (LRPS) and *Lycium ruthenicum* Murr. anthocyanins (LRAC) were comprehensively investigated in this study. LPRS was obtained by water extraction and alcohol precipitation and further purified using diethylaminoethyl cellulose (DEAE‐Cellulose) and Sephadex G‐75 columns. High‐performance liquid chromatography (HPLC) and Fourier transform‐infrared (FT‐IR) spectroscopy were used to characterize the purified LRPS. The results showed that the purified LRPS contained heteropolysaccharides, mainly composed of arabinose, galactose, and glucose with weight percentage of 41.2%, 33.6%, and 10.8%, respectively. More importantly, LRPS (500 μg/ml) and LRAC (80 μg/ml) failed to impede the proliferation of tumor cells when applied solely (48 h incubation), yet remarkable antineoplastic effects were found once they were applied altogether, since the LoVo cells, a typical human colorectal carcinoma cell line, were significantly inhibited by the mixture of LRPS (150 μg/ml) and LRAC (20 μg/ml) (LRPS&AC) in 24 h. The antineoplastic activity resulted from the combination of both LRPS and LRAC (LRPS&AC), by means of blocking the cell cycle at the G0–G1 phase and inducing LoVo cell apoptosis via reactive oxygen species (ROS)‐dependent pathway. The inhibitory effects of LRPS&AC were specific to the tumor cells, without imposing on the proliferation of normal cells. Western blotting revealed that the antitumor effect was related to the mitochondria‐mediated apoptosis launched by the cross‐action of PI3K/Akt (phosphatidylinositol 3‐kinase/protein kinase B) and JAK2/STAT3 (janus kinase 2/signal transduction and activator of transcription 3) signaling pathways. These findings for the first time reveal the synergistic antitumor effects of LRPS&AC and the related mechanisms, which enable *Lycium ruthenicum* Murr. to serve as a natural source to develop therapeutic reagents and functional foods with antineoplastic properties.

## Graphical abstract

*Lycium ruthenicum* Murr. polysaccharides (LRPS) and *Lycium ruthenicum* Murr. anthocyanins (LRAC) failed to inhibit tumor cells when used solely, yet remarkable antineoplastic effects were found once they were applied altogether. Blocking the cell cycle, enhancing intracellular reactive oxygen species (ROS), and activating mitochondria‐mediated apoptosis by the interaction of PI3K/Akt (phosphatidylinositol 3‐kinase/protein kinase B) and JAK2/STAT3 (janus kinase 2 (JAK2)/signal transduction and activator of transcription 3) signaling pathways are involved in the antitumor effects. The findings reveal for the first time that the combination of LRPS and LRAC could inhibit tumor cells and the related mechanisms.

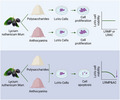

## 1 INTRODUCTION

Colorectal cancer, featuring with uncontrollable proliferation of carcinoma cells on the inner lining of rectum or colon, ranks third among all cancers in terms of incidence and second in terms of mortality (Siegel et al., 2020). Every year, more than 1.2 million new cases were reported along with 0.6 million deaths around the world (Brenner et al., 2014; Siegel et al., 2020). In China, due to the sedentary lifestyle and westernized diet driven by the country's rapid‐growing economy, the incidence of colorectal cancer among 100,000 individuals has increased from 16.8 cases to 20.7 cases (2011 to 2020), with an ever‐growing rate as predicted up until 2025 (Zhang et al., 2019; Zhu et al., 2017). Traditionally, chemosynthetic drugs such as capecitabine, docetaxel, and oxaliplatin were used to inhibit the progression of the cancer (Drott et al., 2018; Schuller et al., 2000; Fan et al., 2015). However, neutropenia, myelosuppression, and neurotoxicity are observed during the treatment using these chemical reagents (Hamauchi et al., 2017). Apart from that, immunosuppression, inhibition of normal cells, and nontarget cell killing for unraveled mechanisms are still looming large on the application of the chemotherapies against human colorectal cancer.

*Lycium ruthenicum* Murr., known for its purple ellipsoid fruit, is a wild thorny shrub growing in the salinized desert of northwestern China. The fruit of *L. ruthenicum*, also called “black goji berry,” have long been recorded in Tibetan medical classic Jing Zhu Ben Cao as herbal medicine and functional food (Zheng et al., 2011). An ever‐growing body of scientific research has found that the berries of *L. ruthenicum* possess versatile biological activities, such as combating fatigue, ameliorating heart attacks, modulating gut microbiota, and relieving immunosuppression (Gong et al., 2015; Wu et al., 2016). *L. ruthenicum* diet and its anthocyanin were also demonstrated with favorable functions on gut microbiota and neuronal cells (Tang et al., 2017; Tian et al., 2019). *Lycium barbarum*, another member *Lycium* genus, closely related to *L. ruthenicum*, has been reported with antineoplastic activities against cancers rooted in liver, bladder, and colon (Shin et al., 2011; Zhang et al., 2013; Zhang et al., 2017). The polysaccharides and the anthocyanins from *Lycium barbarum* have been proved to be the main bioactive compounds inhibiting growth of the carcinoma cells, suggesting that the counterparts from *L. ruthenicum* could also have some antineoplastic activities. However, the effects of *L. ruthenicum* on tumor cells have yet to be deeply investigated, and the mechanisms behind the biological activities remain poorly understood.

The present study was therefore designed to isolate bioactive compounds from *L. ruthenicum*, to dissect their cytotoxicity against human colorectal carcinoma LoVo cells. The results show that, *L. ruthenicum* polysaccharides (LRPS) and *L. ruthenicum* anthocyanins (LRAC) have a synergistic antineoplastic effect on the carcinoma cells, without lowering the viability of normal immune cells. The mechanism behind this synergistic antineoplastic effect lay in specifically arresting the cancer cell progression at G0–G1 phase, inducing the cell apoptosis via reactive oxygen species (ROS)‐dependent pathway, and mediating the cross‐action between PI3K/Akt (phosphatidylinositol 3‐kinase/protein kinase B) and JAK2/STAT3 (janus kinase 2 (JAK2)/signal transduction and activator of transcription 3) signaling pathways. These findings revealed for the first time that the anticarcinoma effect of *L. ruthenicum* was contributed by the combination of LRPS and LRAC, suggesting that natural products from *L. ruthenicum* could be used to develop anticancer drugs and functional foods with prophylactic effects against colorectal cancer.

## 2 MATERIALS AND METHODS

### 2.1 Chemicals and reagents

The fresh *L. ruthenicum* berries were collected from the Shenmu area located on the Loess Plateau in the northwest of China. To avoid breakdown of the anthocyanins, the berries were sun‐dried and stored at −18°C for future use. AB‐8 macroporous resin was obtained from Xinhu Chemicals Co. Ltd. All the monosaccharides (D‐mannose, D‐ribose, L‐rhamnose, D‐glucose, D‐xylose, D‐galactose, D‐glucuronic acid, D‐galacturonic acid, L‐arabinose, and D‐fucose) serving as the standards to characterize LRPS were purchased from Sigma‐Aldrich. Pectinase, propidium iodide (PI), and 3‐(4,5‐dimethylthiazol‐2‐yl)‐2,5‐diphenyltetrazolium bromide (MTT) were provided by Solarbio Biotech Co. Ltd. Nanjing Jiancheng Bioengineering Co. Ltd. provided 2,7‐dichlorofluorescein diacetate (DCFH‐DA). All cell culture reagents were purchased from Sinopharm. Primary antibodies to PI3K, phosphorylated Akt (p‐Akt), phospho‐JAK2 (p‐JAK2), phospho‐STAT3 (p‐STAT3), BCL2 associated X protein (Bax), B‐cell lymphoma 2 (Bcl‐2), Caspase‐3, and β‐actin for Western blot analysis were purchased from Cell Signaling Technology, Inc. (Beverly, USA). All the other chemicals were of the highest grade available and used without further purification.

### 2.2 Preparation of polysaccharides from *L. ruthenicum*

The polysaccharides from *L. ruthenicum* (LRPS) were prepared according to the reported protocol with modification to eliminate the pectin (Zhu et al., 2013). First, the dried fruit of *L. ruthenicum* was ground into powder, 150 g of which was dissolved in 500 ml saline sodium citrate (SSC) buffer (pH = 3.0) containing 0.4% pectinase (w/v). The mixture was placed in a shaking bath (100 r/min) at 65°C for 2 h. After extraction, the solution was collected by vacuum filtration, and the residue was re‐extracted two times using the same procedure. All the extraction solutions containing LRPS were combined and concentrated on a rotary evaporator at 45°C for 1 h. Subsequently, ethanol (90%, v/v) of fourfold volume was slowly added to the concentrated solution to precipitate the polysaccharides. The sediment was redissolved and treated with Sevag reagent to remove protein fractions, followed by dialysis against distilled water for 48 h. The deproteinized solution was concentrated again and subjected to vacuum freeze‐drying to obtain crude LRPS (CLRPS). The CLRPS was further purified using DEAE‐Cellulose and Sephadex G‐75. The detailed procedure can be found in Appendix S1. The purified LRPS4 was subjected to freeze‐drying, and the resulting LRPS of high purity was obtained and stored for future use.

### 2.3 Extraction and purification of anthocyanins from *L. ruthenicum*

The extraction of *L. ruthenicum* anthocyanins (LRAC) was performed according to the previous study with slight modification (Yan et al., 2018). Briefly, 10 g of *L. ruthenicum* fruit powder was added to 400 ml of 75% ethanol (v/v) containing 0.1% formic acid. The resulting mixture was kept in a water bath at 45°C for 4 h, followed by filtration using a tammy cloth. The extract was then concentrated using a vacuum rotary evaporator at 45°C for 1 h. After that, the concentrated extract was loaded on an AB‐8 macroporous resin column (5 × 30 cm), followed by washing with deionized water (1.5 times of bed volume) to carry away the constituents of strong polarity. The anthocyanin constituent was then eluted from the column with 75% ethanol (v/v) at the flow rate of 2.5 ml/min. Finally, the eluate was combined and concentrated by lyophilization to obtain the purified LRAC. The LRAC was in powder form and stored at 4°C for follow‐up experiments. The LRAC was then characterized by high‐performance liquid chromatography (HPLC), and the detailed procedure was shown in Appendix S1.

### 2.4 Analysis of monosaccharide constitution of LRPS by HPLC

The LRPS of 20 mg was hydrolyzed to monomer by 2 ml of trifluoroacetic acid (TFA) (3 M) at 95°C for 8 hr in a sealed ampoule (10 ml) under nitrogen protection. Analysis of the monosaccharides was performed according to the previous method (Liang et al., 2020). Briefly, 100 μL of the hydrolyzed LRPS was dissolved in 300 μL NaOH (0.3 M), followed by the addition of 200 μL of methanol solution with 1‐phenyl‐3‐methyl‐5‐pyrazolone (PMP) (0.5 M), which reacts with the monosaccharides for enhanced ultraviolet (UV) signal. The reaction was performed at 70°C for 60 min and terminated by 200 μL of HCl (0.3 M). The resulting solution was extracted with 2 ml chloroform three times, and the aqueous layer was filtered through a 0.45‐μm membrane for HPLC analysis. A LC‐2010A HPLC system (Shimadzu, Japan) equipped with a RP‐C_18_ analytical column (4.6 mm i.d. × 250 mm, 5 μm, Venusil, USA) was used for analysis. HPLC‐grade acetonitrile served as mobile phase A, and the mobile phase B was comprised of KH_2_PO_4_ (3.3 mM), triethylamine (3.7 mM), and 10% acetonitrile. Gradient elution was applied using linear decrease of phase B with the proportion of 97%–95%–92%–90% during 0–6–8–40 min. The flow rate of 1.0 ml/min was applied for elution and the detection was carried out at a wavelength of 250 nm. All the separations and analysis were performed at ambient temperature (25°C).

### 2.5 Characterizing LRPS by FT‐IR

Fourier transform infrared (FT‐IR) spectroscopy (Bruker) was used to characterize LRPS. The prepared LRPS were pulverized with KBr (infared [IR] grade) to obtain tablets for IR analysis. IR signals were recorded from the accumulation of 32 scans in the range from 4000 cm^−1^ to 500 cm^−1^ with the resolution of 0.1 cm^−1^. To minimize the background noise, spectral scan of samples was repeated at least three times.

### 2.6 Antitumor activity of LRPS and LRAC

#### 2.6.1 Cell lines and culture

LoVo cells, a typical human colorectal carcinoma cell line, were provided by the Cell Bank of Institute of Biochemistry and Cell Biology, Chinese Academy of Sciences. The cells were cultured in Roswell Park Memorial Institute (RPMI)‐1640 medium supplemented with 10% heat‐inactivated fetal bovine serum (FBS), 100 U/ml penicillin, and 100 µg/ml streptomycin in a humidified incubator at 37°C with 5% CO_2_. All the assays on LoVo cells were independently performed in triplicate.

#### 2.6.2 Cell viability

The MTT assay was used to evaluate the viability of LoVo cells treated with LRPS, LRAC, or altogether. Briefly, 100 µl of medium containing cells of 1 × 10^5^ in was seeded in a well on the 96‐well microplate. When cells were grown to 70% confluence, LRPS of serial concentrations (150 µg/ml, 300 µg/ml, and 500 µg/ml) with or without LRAC (20 µg/ml) were added. In the control groups, the same volume of PBS was added instead. The fluorouracil (5‐FU) of 100 µg/ml was also introduced to serve as the positive control. After 24 h or 48 h, 10 µl of MTT (5 mg/ml) in PBS solution was added to each well to the final concentration of 0.5 mg/ml, followed by incubating the plate at ambient temperature for another 4 h. After that, the media containing MTT were discarded, prior to adding 100 µl of solution (pH = 4.8) containing HCl (10 mM), sodium dodecyl sulfate (SDS) (10%), and isobutyl alcohol (5%) to each well and mixing thoroughly to dissolve the formed crystal formazan. After the overnight incubation at 37°C, the absorbance was recorded at 570 nm. Cell viability was expressed as a percentage of absorbance values of the treated cells to that of the control cells.

#### 2.6.3 Cytotoxicity assessment by the LDH assay

Cytotoxicity was evaluated by the lactate dehydrogenase (LDH) assay after the treatment with the combination of LRPS and LRAC (LRPS&AC). Cells were subjected to the same processing as in the cell viability assay. After that, 120 μl of culture supernatants was pipetted and subjected to LDH analysis according to the operation manual of the kit. Finally, absorbance at 450 nm on the ELISA reader (Rayto‐RT 6000) was recorded. The cytotoxicity was expressed as the LDH leakage ratio of the treated group to the control group.

#### 2.6.4 Flow cytometric analysis of cell cycle distribution

Cell cycle distribution of the LoVo cells was determined by measuring the content of double‐stranded DNA (biomarker) using PI staining. Briefly, the treated LoVo cells were seeded in 6‐well culture plates for 24 h. Then trypsin–EDTA (ethylenediaminetetraacetic acid) was used to detach the adherent cells, prior to washing the dissociative cells twice with PBS. After that, suspensions were subjected to a low‐speed centrifugation, by removing the supernatants, and the cell pellets were obtained. The cell pellets were resuspended and fixed with 1 ml of 70% ethanol at −20°C overnight, washed by PBS, lysed, and incubated with PI and ribonuclease (RNase) for 30 min in dark at room temperature. The cell cycle distribution was analyzed using FACS Calibur flow cytometry (Becton Dickinson). The result was analyzed and visualized using ModFit LT 5.0‐Flow Cytometry Modeling (Verity Software House).

#### 2.6.5 Flow cytometric analysis of apoptosis

Cells for apoptosis analysis were treated according to the procedure as described in cell cycle analysis section. An apoptosis detection kit (BestBio) was employed to analyze apoptotic distribution of the cells. PS (phosphatidylserine) usually found on the inside of the cell membrane and nuclear DNA were used as the biomarkers for the assay. Simultaneous quantification of vital and apoptotic cells was achieved by using dual dying (Annexin V‐FITC (fluorescein isothiocyanate) and PI, targeting PS and nuclear DNA, respectively). Briefly, cells of 10^6^ were collected and washed twice with PBS, followed by being resuspended in 400 µl of binding buffer with the addition of Annexin V‐FITC (5 µl) and PI (10 µl). The resulting mixtures were kept in dark at 4°C for 10 min, and the cells were loaded on the flow cytometer for analysis. The result was analyzed and visualized using CellQuest‐Flow Cytometry Software (Becton Dickinson).

#### 2.6.6 Analysis of intracellular ROS in LoVo cells

DCFH‐DA, a cell‐permeable fluorescent probe, was used to determine the intracellular ROS levels of the LoVo cells. The cells were cultured at a density of 10^5^/well on 24‐well plates for 24 h to allow adhesion. After that, cell media were substituted with serial concentrations of LRPS containing LRAC (20 µg/ml), or H_2_O_2_ designated as positive control. The cells were then incubated for 24 h, followed by PBS washing of two times, and incubated with DCFH‐DA probe (10 µM) at 37°C for 20 min. The fluorescence intensity generated by DCFH‐DA in the LoVo cells was recorded by the flow cytometry (excitation: 485 nm, emission: 525 nm). The result was analyzed and visualized using CellQuest‐Flow Cytometry Software (Becton Dickinson).

### 2.7 Western blotting

Western blotting was carried out according to the previous report with modifications (Zhao et al., 2019; Shi et al., 2014). The expression levels of PI3K, p‐Akt, p‐JAK2, p‐STAT3, Bax, Bcl‐2, Caspase‐3, and β‐actin after the treatments were measured. Briefly, LoVo cells (1 × 10^6^ cells/well) were seeded in a six‐well plate, followed by incubation overnight at 37°C with 5% CO_2_. Then the cells were treated for 24 h with LRPS, LPAC, and LRPS&AC, respectively. After that, cytoplasmic and nuclear protein were extracted using NE‐PER Nuclear and Cytoplasmic Extraction Reagents (Thermo Scientific, Rockford, USA) according to the operation manual. Sodium dodecyl sulfate–polyacrylamide gel electrophoresis (SDS‐PAGE) was used to separate the proteins, which were then transferred to polyvinylidene difluoride (PVDF) membranes (Millipore). The loaded membranes were blocked with 5% skimmed milk in Tris‐buffered saline with Tween‐20 (TBST) buffer for 1 h at room temperature, followed by incubation with the primary antibodies at 4°C on a shaker overnight. The membranes were then washed twice and incubated at room temperature for 1 h with the corresponding secondary antibody conjugated with horseradish peroxidase. The blots were detected by enhanced chemiluminescence (ECL) detection kits (Bio‐Rad, Hercules, USA) and analyzed using Empiria Studio^®^ software.

### 2.8 Statistical analysis

All the treatments in each assay were carried out independently in triplicate, and the data were expressed as mean ± SD. Significant difference between groups was assessed by one‐way analysis of variance (ANOVA) using SPSS 18.0 software, which was also used for multiple comparisons and Duncan's multiple range tests. The difference was considered significant if *p* < .05, and extremely significant if *p* < .01.

## 3 RESULTS AND DISCUSSION

### 3.1 Characterization of LRPS by HPLC and FT‐IR

**FIGURE 1:**
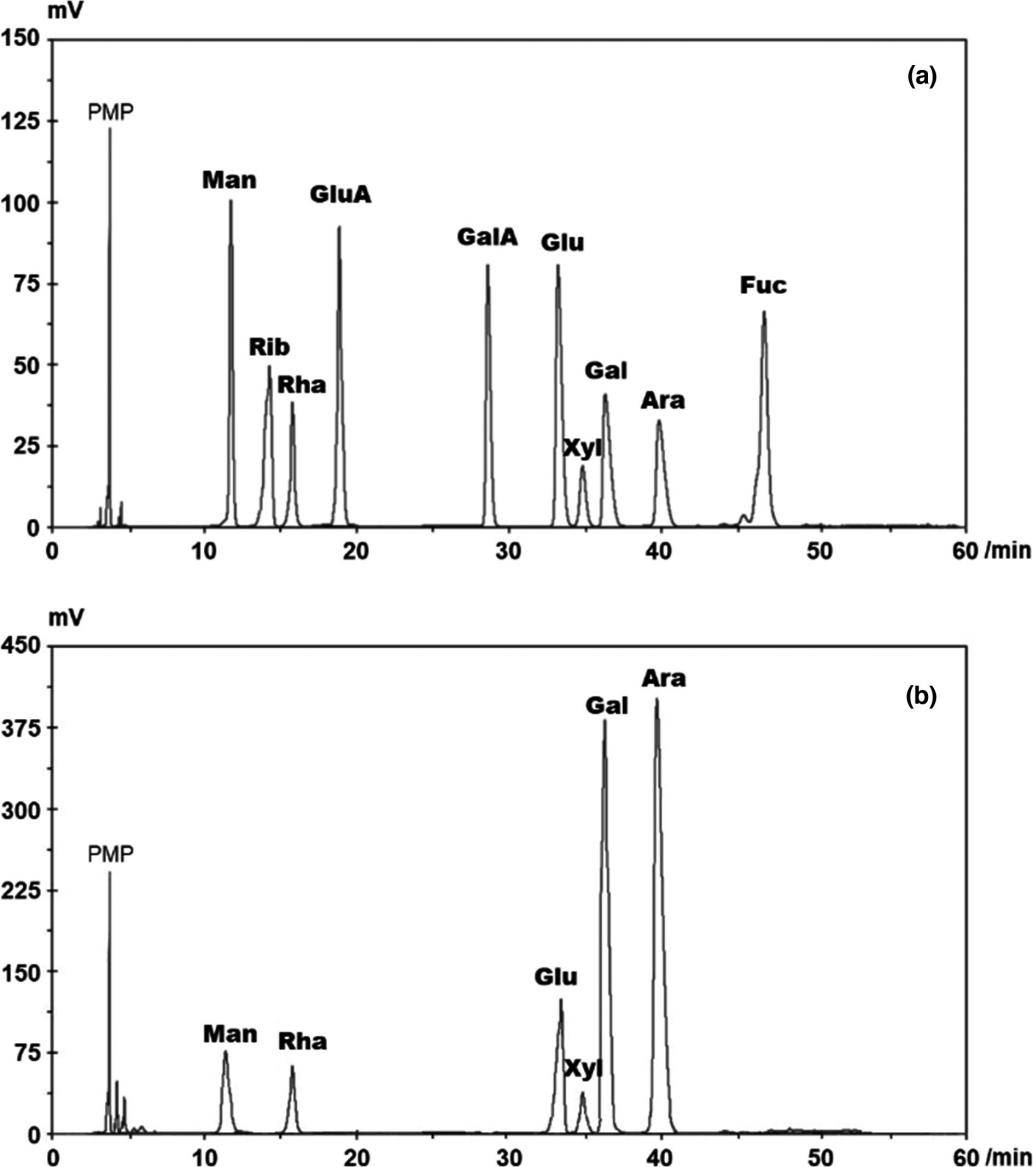
Analysis of the monosaccharide constitution of *Lycium ruthenicum* Murr. polysaccharides (LRPS) by high‐performance liquid chromatography (HPLC). (a) Elution chromatograms of 10 1‐phenyl‐3‐methyl‐5‐pyrazolone (PMP)‐labeled standard monosaccharides. (b) The PMP‐labeled monosaccharides released from LRPS. Monosaccharide peaks from left to right: D‐Mannose, L‐ribose, D‐rhamnose, D‐glucuronic acid, D‐galacturonic acid, D‐glucose, D‐xylose, D‐galactose, L‐arabinose, D‐fucose (Internal Standard)

Here, water extraction and repeated ethanol precipitation were used to extract polysaccharides from *L. ruthenicum* (LRPS). The crude LRPS was further purified by DEAE‐Cellulose and Sephadex G‐75, and the results are shown in Figure S1 (Appendix S1). To characterize the chemical constitution of LRPS, the PMP‐derived HPLC method established in our previous work was applied with slight modifications (Liang et al., 2020). As shown in Figure 1a, 10 PMP‐labeled monosaccharides serving as the standards were distinctly separated within 60 min, as well as the constituent monosaccharides released from LRPS (Figure 1b). By comparing the retention times of unknown peaks to that of the standard monosaccharides, six monosaccharides including mannose, rhamnose, glucose, xylose, galactose, and arabinose were identified in LRPS, and no uronic acid was detected. The result indicated that LRPS were typical heteropolysaccharides, which were consistent with the previous report (Lv et al., 2013). The LRPS was composed of mannose, rhamnose, glucose, xylose, galactose, and arabinose with the weight percentages of 6.2%, 5.4%, 10.8%, 2.7%, 33.6%, and 41.2%, respectively. Among these monosaccharides, galactose and arabinose accounted for more than 70% content. This agrees with the previous study demonstrating that galactose and arabinose were dominant monosaccharides in the sugar content of *L. ruthenicum* (Peng et al., 2012).

**FIGURE 2:**
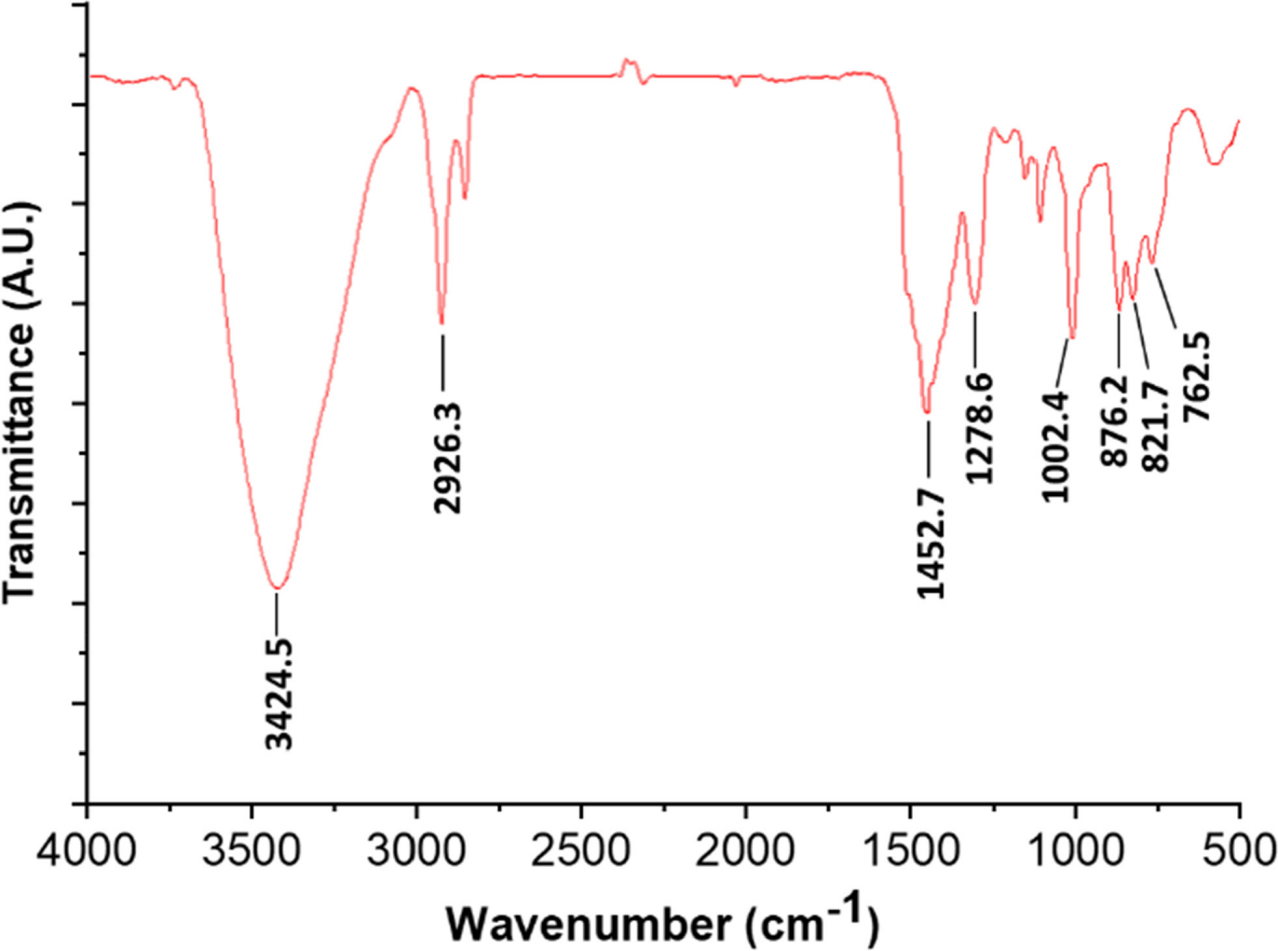
Fourier transform‐infrared (FT‐IR) spectrum of polysaccharides extracted from *Lycium ruthenicum* in the wave number ranging from 4000 to 500 cm^−1^. Characteristic peaks are marked with exact wave numbers of IR adsorption

Fourier transform‐infrared was also applied here to analyze the functional groups in LRPS. As shown in Figure 2, a broad and intense peak at around 3424.5 cm^−1^ was attributed to the O–H stretching vibration of hydroxyl group, a typical moiety in polysaccharides from plants. The band at 2926.3 cm^−1^ was attributed to the stretching vibrations of C–H on CH_2_. The absorption peaks at 1452.7 cm^−1^ and 1278.6 cm^−1^ were caused by the angle vibration and symmetrical deformation of COH groups. A peak at 1093.6 cm^−1^ representing the presence of furanose ring was observed, which could be ascribed to the presence of arabinose in LRPS (Yan et al., 2018). The absorption peaks between 1002.4 cm^−1^ and 876.2 cm^−1^ indicated that most of the monosaccharides in LRPS were of the pyranose type (Liu et al., 2015). The absorption peaks at 821.7 cm^−1^ and 762.5 cm^−1^ suggested that the pyranoses were of both α‐ and β‐configuration (Liu et al., 2015). Therefore, the result of FT‐IR analysis suggested that LRPS were neutral polysaccharides with functional groups.

### 3.2 Synergistic antineoplastic potential of LRPS&AC

#### 3.2.1 Inhibitory effects of LRPS&AC on LoVo cells

**FIGURE 3:**
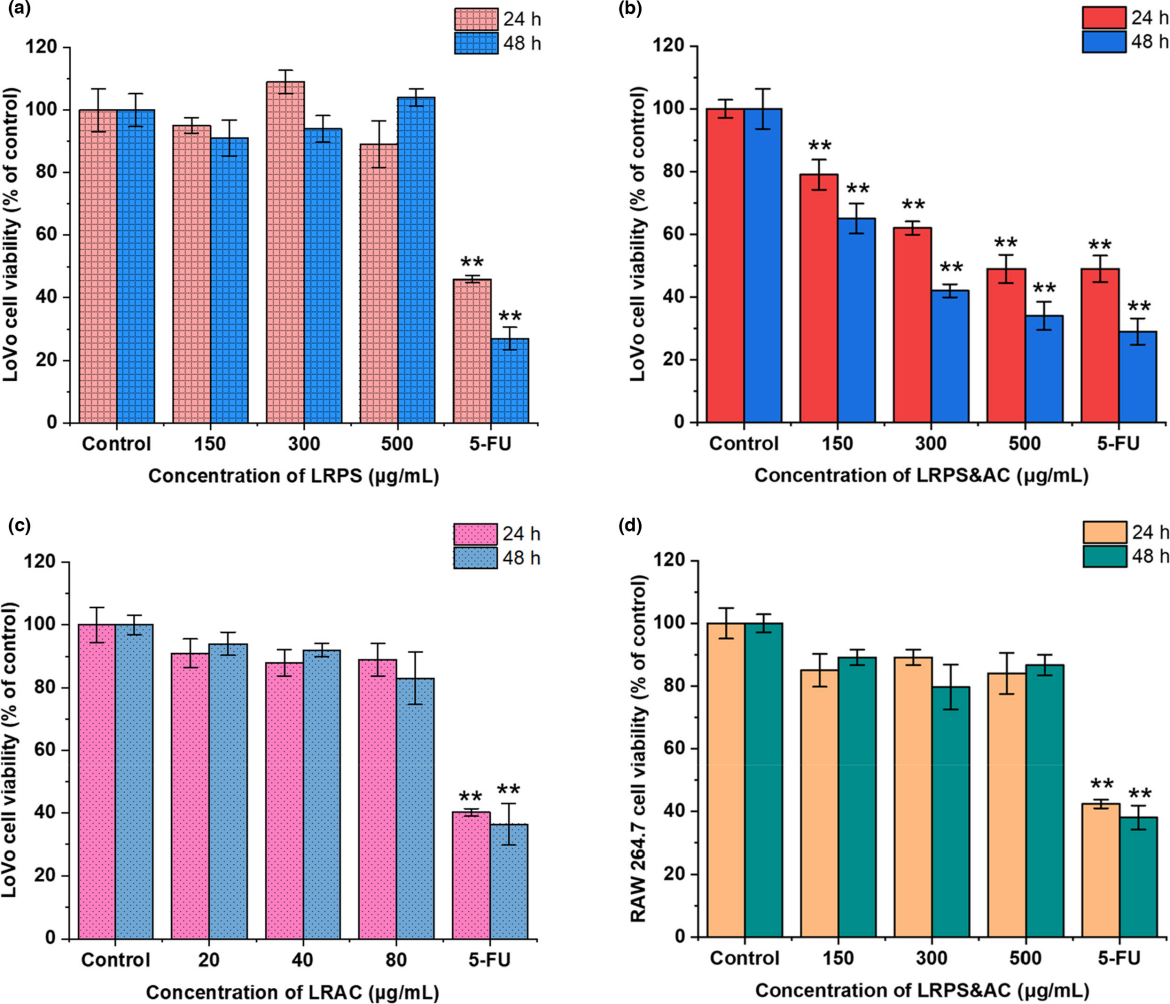
Effects of *Lycium ruthenicum* Murr. polysaccharides (LRPS) and *Lycium ruthenicum* Murr. anthocyanins (LRAC) on the viability of LoVo and RAW 264.7 cells. (a) Result of the 3‐(4,5‐dimethylthiazol‐2‐yl)‐2,5‐diphenyltetrazolium bromide (MTT) assay on LoVo cells treated with LRPS varying in concentration. (b) Synergistic antineoplastic effect of LRPS and LRAC on human colon cancer LoVo cells. Concentration of LRPS was used to represent the amount of LRPS&AC mixture (the combination of LRPS and LRAC), which contained constant LRAC of 20 μg/ml. The cell viability was measured by the MTT assay and expressed with rates versus that of control. (c) Cell viability of LoVo treated with LRAC varying in concentration. (d) Cell viability of RAW 264.7 when treated with LRPS&AC. The same amounts of LRPS&AC incubated with LoVo cells were applied. All the data are expressed as the mean ± SD of three independent experiments. The fluorouracil (5‐FU) of 100 μg/ml was used as the positive control. *p < .05 and **p < .01 indicate a significant difference versus the control group

As inspired by the antineoplastic effects of polysaccharides from *L. barbarum* demonstrated previously (Luo et al., 2009; Zhang et al., 2015; Zhu & Zhang, 2013), we first evaluated the viability of cancer cells solely treated with LRPS. LoVo cells, a typical human colorectal carcinoma cell line, were cultured with LRPS (150 μg/ml–500 μg/ml) for 24 h and 48 h, then the cell viability was measured by the MTT assay. Unfortunately, as shown in Figure 3a, no inhibitory effects were observed when the LoVo cells were incubated with LRPS. Even higher concentrations (up to 1200 μg/ml) and prolonged incubation time (up to 72 h) failed to display any cytotoxicity on the cells (Figure S2, Appendix S1). However, when LRAC (characterized by HPLC as shown in Figure S3, Appendix S1) was introduced, the LoVo cells were significantly inhibited by the combination of LRPS and LRAC (LRPS&AC). As shown in Figure 3b, for incubation of 24 h, the cell viabilities were dose‐dependently reduced down to 87.2%, 83.5%, and 72.7%, respectively. A similar result was also observed with the incubation of 48 h, where LRPS&AC exerted a stronger inhibitory effect (46.7%) comparable to that of 5‐FU, a potent anticancer reagent. To rule out the possibility that the inhibitory effect was caused by LRAC, the cell assay using LRAC alone was carried out. The result showed that LRAC (20 μg/ml to 100 μg/ml) was incapable of reducing viability of the LoVo cells (Figure 3c), suggesting that the inhibitory effect on tumor cell was caused by the combined action of LRPS and LRAC. To confirm the inhibitory effects of LRPS&AC on LoVo cells, LDH assay was performed (Figure S4, Appendix S1). LDH in LoVo cell medium increased with the increment of LRPS&AC (150 μg/ml to 500 μg/ml), suggesting that LRPS&AC was of cytotoxic effect on tumor cells by inducing membranolysis. This result corroborated that LRPS&AC was of antineoplastic potentials on LoVo cells. The inhibitory effect could be ascribed to the irreversible cell member damage caused by LRPS&AC, although the exact molecular mechanism requires in‐depth investigations.

As many antineoplastic chemicals were reported with undesirable immunosuppression effects (Cho et al., 2015), we hence examined the influence of LRPS&AC on RAW 264.7, a typical immune‐functional cell line. As shown in Figure 3d, when treated with LRPS&AC varying in concentration (150 μg/ml to 500 μg/ml), RAW 264.7 cells displayed no significant reduction of viability, indicating that LRPS&AC did not interfere with the growth of immune cells. Besides, HepG2, a human hepatoma cell line, was also observed with the stunted growth when treated with LRPS&AC (Figure S5, Appendix S1), suggesting that cytotoxicity of LRPS&AC was not limited to one type of carcinoma cell. All the results shown above demonstrated that LRPS and LRAC had a synergistic anticarcinogenic effect on tumor cells. The LRPS&AC selectively inhibited the cancer cells, without lowering the viability of normal cells, a property that enabled its application as a natural antineoplastic reagent to prevent colorectal‐related cancers with minimal side‐effects. Several investigations reported the antineoplastic effect of polysaccharides' natural plant, including *L. barbarum*, a sibling specie of *L. ruthenicum* (Zhang et al., 2005, 2013, 2017). Therefore, we proposed that LRPS, as heteropolysaccharides with rich tertiary structures, are mainly responsible for recognizing specific glycoproteins on tumor cells' surface, leading to inhibited proliferation of the carcinoma cells with the aid of LRAC (Li et al., 2021; Sharon & Lis, 1993).

#### 3.2.2 Induced cell cycle arrest of LoVo cells by LRPS&AC

**FIGURE 4:**
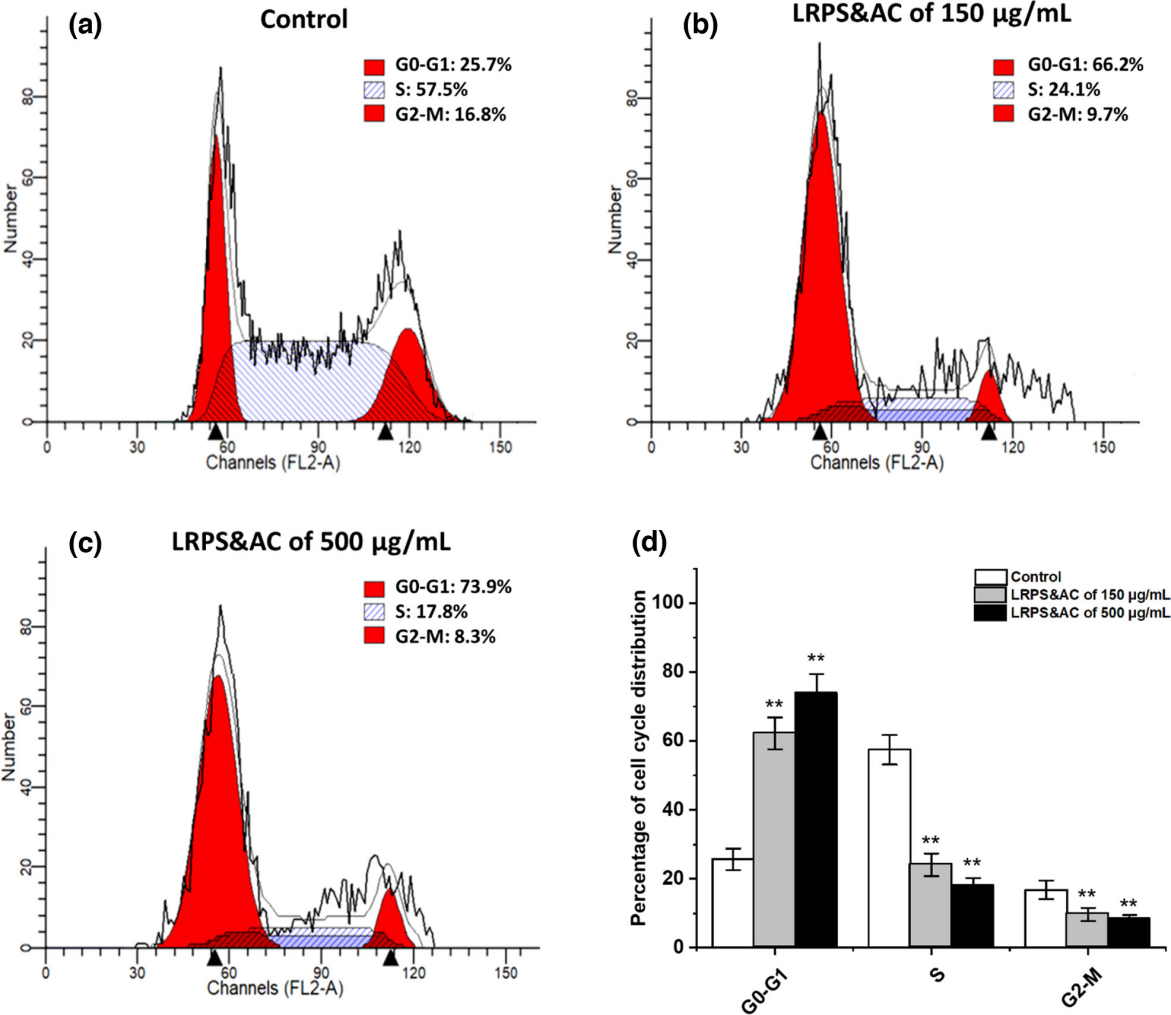
LRPS&AC (*Lycium ruthenicum* Murr. polysaccharides and anthocyanins, the combination of LRPS and LRAC) inhibited the proliferation of LoVo cells by interfering with the cell cycle distribution. (a) Control (LRPS&AC was replaced with the same volume of medium). (b) Cells treated with 150 μg/ml of LRPS&AC. (c) Cells treated with 500 μg/ml of LRPS&AC. (d) Percentage of cell populations in G0–G1, S, and G2–M phases. Concentration of LRPS was used to represent the amount of LRPS&AC mixture, which contained constant LRAC of 20 μg/ml. Data were shown as the means ± SD (*n* = 3), and asterisks denote a statistical significance in comparison to that of the control group (*p < .05, **p < .01)

Analysis of cell cycle distribution was carried out to determine whether the inhibitory effect on the tumor cells was due to the blockade of cell cycle progression by the synergistic effect of LRPS and LRAC. As shown in Figure 4, when treated with low dosage (150 μg/ml) and high dosage (500 μg/ml) of LRPS&AC, cells in S phase were significantly reduced to 24.1% and 17.8%, comparing to that of 57.5% in the control group, while cells in the G0–G1 phase were observed with dramatic increase, accounting for 66.2% and 73.9%. This indicated that LRPS&AC was able to seize the cell progression at the G0–G1 phase, probably by interfering with the initiation of DNA and histone synthesis. As a result, LoVo cells failed to produce replicated genetic materials that are prerequisite for mitosis, leading to impeded proliferation of the cancer cells (Choi et al., 2019). Previous studies have demonstrated that the antiproliferative activities of natural polysaccharides were associated with arresting cancer cells at S phase (Luo et al., 2009; Wang et al., 2018; Zhong et al., 2013). Here, LRPS&AC inhibited LoVo cells' replication by G0–G1 arrest, which coincided with earlier researches elucidating anthocyanins from black currant and purple‐shoot tea cells were able to block cancerous cells at G0–G1 phage (Hsu et al., 2012; Nanashima et al., 2017). Therefore, we proposed that LRAC was the dominant factor to interrupt the cell cycle progression by acting on particular signaling pathways different from that of natural polysaccharides (Huang et al., 2008).

#### 3.2.3 Effects of LRPS&AC on LoVo cell apoptosis

**FIGURE 5:**
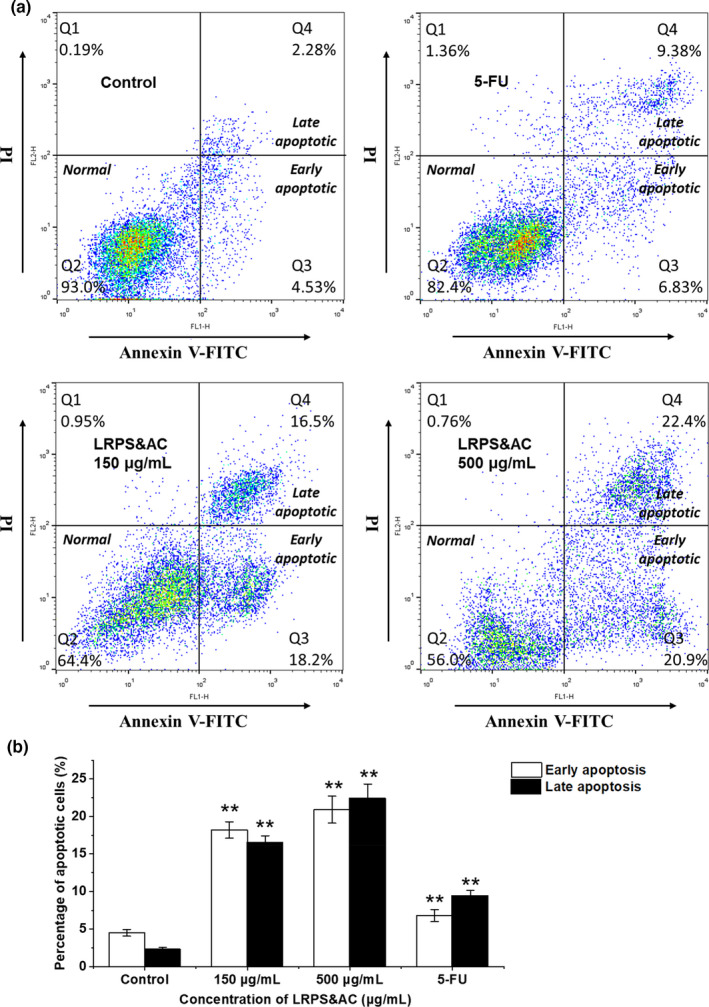
Effect of LRPS&AC (*Lycium ruthenicum* Murr. polysaccharides and anthocyanins, the combination of LRPS and LRAC) on the induction of LoVo cell apoptosis. (a) Typical apoptosis plot of LoVo cells treated with LRPS&AC varying in concentration. Annexin V‐FITC (fluorescein isothiocyanate) and propidium iodide (PI) double staining was used to measure the apoptosis of LoVo cells. Threshold values for the two fluorescent channels were designated as 10^2^ FI (fluorescence intensity). Cell counts with FI higher than 10^2^ were considered as Annexin V‐FITC positive (Annexin V‐FITC+) or PI positive (PI+). (b) Percentage of LoVo cells in early and late apoptotic stages as presented in (a). Concentration of LRPS was used to represent the amount of LRPS&AC mixture, which contained constant LRAC of 20 μg/ml. The results were presented by mean ± SD (*n* = 3). Asterisks denote a significant difference versus that of the control group (*p < .05, **p < .01)

Apoptosis is an intrinsic biological mechanism to obliterate dysfunctional cells. There is mounting evidence suggesting that the induced cell apoptosis is involved in antineoplastic effects of natural products (Daniel et al., 2006; Fulda, 2010; Plitzko et al., 2017). Therefore, Annexin V‐FITC and PI double staining assay was employed here to examine if apoptosis is a possible process for LRPS&AC‐induced LoVo cell death. As can be seen in Figure 5a, more than 90% of LoVo cells were detected with Annexin V‐FITC‐/PI‐ when no LRPS&AC was induced (control group), indicating that apoptosis of LoVo cells barely occurred without LRPS&AC. When treated with LRPS&AC of 150 μg/ml, cells in early apoptotic stage (Annexin V‐FITC+/PI‐) and late apoptotic stage (Annexin V‐FITC+/PI+) significantly raised to 18.2% (*p* < .01) and 16.5% (*p* < .01), comparing with that of the control group. Furthermore, when the amount of LRPS&AC increased to 500 μg/ml, more than 43.0% of cells were observed with apoptotic characteristics (20.9% in early apoptotic stage, 22.4% late apoptotic stage), indicating that the induced cell apoptosis by LRPS&AC was carried out in a dose‐dependent manner. The results suggested that LRPS&AC was able to induce tumor cell apoptosis by initiating the progress of the programmed cell suicide. Comparing with 5‐FU, a potent anticancer chemical, LRPS&AC showed a higher apoptosis‐inducing capability on LoVo cancer cells (Figure 5b). This was consistent with the analysis of cell cycle distribution, confirming that the LRPS&AC was able to inhibit the proliferation of LoVo cells. When tumor cells are in the progress of growth and metastasis, the enhanced rate of proliferation and decreased apoptosis are two core processes (He et al., 2012). An ideal anticancer agent is expected to be able to persistently reverse the two processes at the same time. This was confirmed by the current study, as LRPS&AC was proved with antiproliferative effect on LoVo cells by both arresting cell cycle and inducing apoptosis.

With the increasing dosage of LRPS&AC, LoVo cells both in early‐stage apoptosis and late‐stage apoptosis (necrosis) raised accordingly, suggesting LRPS&AC was of a dose‐dependent inhibitory effect on the tumor cells. Along with the biochemical changes, morphological abnormalities, such as shrinkage, nuclear condensation, and formation of apoptosis bodies, were observed during experimentation (data not shown). This agrees with our previous work revealing that inducing apoptosis and blocking cell cycle at certain phase are two synergetic pathways for natural products to exert antineoplastic bioactivities (Liang et al., 2020; Wang et al., 2018). As observed in this study, LRPS&AC was able to inhibit the growth of cancer cells (LoVo and HepG2), without imposing cytotoxicity on normal immune cells, a property enabling it to be used as a promising natural anticancer reagent with limited side‐effects, although its efficacy in reducing malignancy tumors and cancer‐ridden animals requires further investigations.

#### 3.2.4 Intracellular ROS in LoVo cells treated with LRPS&AC

**FIGURE 6:**
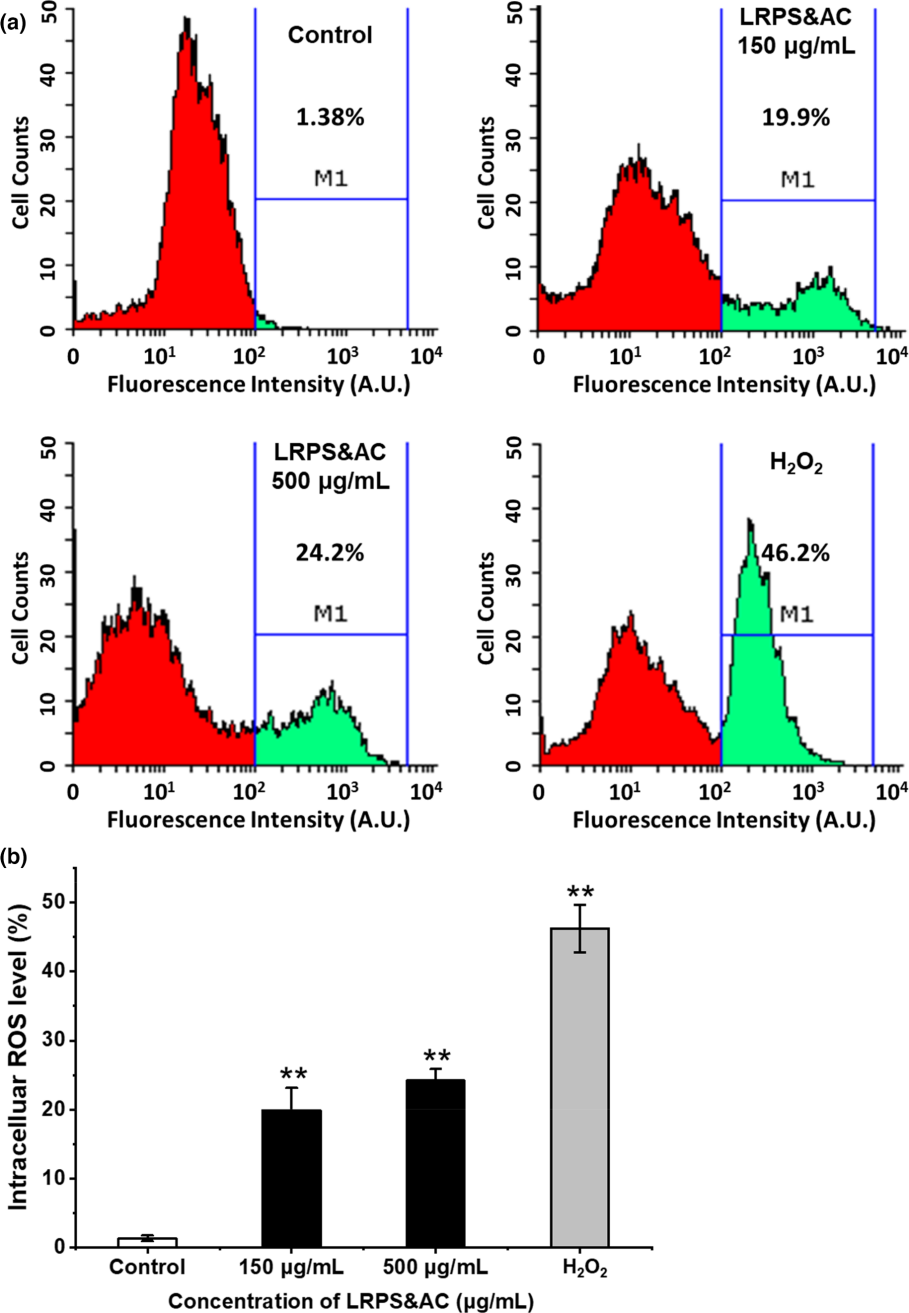
LRPS&AC (*Lycium ruthenicum* Murr. polysaccharides and anthocyanins, the combination of LRPS and LRAC)‐mediated accumulation of intracellular reactive oxygen species (ROS) in LoVo cells. (a) Intracellular ROS in LoVo cells LRPS&AC with high (500 μg/ml) and low (150 μg/ml) concentrations. Cells with fluorescence intensity higher than 10^2^ were considered as significant ROS accumulation (green area). Hydrogen peroxide (H_2_O_2_) was used as positive control. (b) Relative LoVo cell counts with elevated ROS levels as shown in (a). Concentration of LRPS was used to represent the amount of LRPS&AC mixture, which contained constant LRAC of 20 μg/ml. The results were presented by the mean ± SD of three independent experiments. Asterisks denote a significant difference compared with the control group (*p < .05, **p < .01)

Previous studies demonstrated that natural products, such as flavonoids, polyphenols, and polysaccharides, are capable of elevating the ROS level in mitochondria, which in turn push cancer cells to the brink of their toxic threshold (Kaur et al., 2009; Sandoval‐Acuna et al., 2014; Shi et al., 2013). Given that, the inhibitory effect of LRPS&AC on LoVo cells might be related to the induced ROS, disturbing redox homeostasis of the LoVo cells. Here, the DCFH‐DA assay was used to measure the intracellular ROS levels, while H_2_O_2_ served as positive control. Flow cytometry was applied here to detect the fluorescent signals from oxidation of DCFH‐DA by ROS, as well as to sort the cells with different levels of ROS accumulation. As shown in Figure 6a, in the control group, more than 95% of LoVo cells were in normal state since nearly no ROS accumulation was observed. When treated with LRPS&AC of 150 μg/ml, the ROS level in the LoVo cell was significantly increased, as evidenced by cell counts of 19.9% fell into the ROS positive region. More cells of ROS accumulation were detected for 500 μg/ml LRPS&AC treatment (24.2%), indicating that LRPS&AC was able to elevate the ROS level of cancer cells in a dose‐dependent manner. Statistical result of the ROS assay is shown in Figure 6b. Although the cells with accumulated ROS induced by LRPS&AC were lower than that of H_2_O_2_, the escalated oxidative stress could break the redox balance in the LoVo cells, resulting in the programmed cell suicide.

Mitochondria are considered as the main organelle to process intracellular ROS, an upstream factor to regulate subsequent biochemical signals. Previous studies showed that natural products can act on the mitochondria‐mediated ROS elimination (Sreelatha et al., 2011). Therefore, the accumulated ROS in the LoVo cells could be ascribed to abnormality of mitochondria induced by LRPS&AC. In this study, LRPS&AC was able to trigger ROS accumulation in LoVo cells, while the cell apoptosis induced by LRPS&AC was also observed (Figure 6). Several studies reported that ROS served as a second messenger in multiple signaling pathways and played an important role in regulating the activity of certain enzymes involved in programmed cell death (Hengartner, 2000; Juan et al., 2008). LRPS was observed with abilities to scavenge free radicals in vitro (Figure S6, Appendix S1). On the other hand, by cooperating with LRAC, LRPS was able to trigger ROS accumulation in LoVo cells. These results were consistent with a previous report that phytochemicals were of both anti‐ and pro‐oxidant activities (Kaur et al., 2009). Recent advances demonstrated that several phytochemicals, such as THC (tetrahydrocurcumin), RSV (resveratrol), and GIP (*Ganoderma lucidum* polysaccharides), were able to interfere with redox homeostasis of tumor cells (Han et al., 2016; Hu et al., 2019; Miki et al., 2012). The LRPS&AC treatment and ROS generation were proved to be closely associated, and the finding provided a new molecular mechanism underlining the antineoplastic activities of natural products from *L. ruthenicum*. Taken together, LRPS&AC, natural products from *L. ruthenicum*, could be used as the reagents to mitigate carcinogenesis of colorectal cancer. A campus‐based questionnaire showed that, most people considered berries of *L. ruthenicum* as healthy food, and preferred them to *L. barbarum* for premium nutriment and health benefits (Table S1, Appendix S1), suggesting that functional foods developed from *L. ruthenicum* would have vast potential market and wide consumer group.

### 3.3 Effects of LRPS&AC on the expression of apoptosis‐related proteins

**FIGURE 7:**
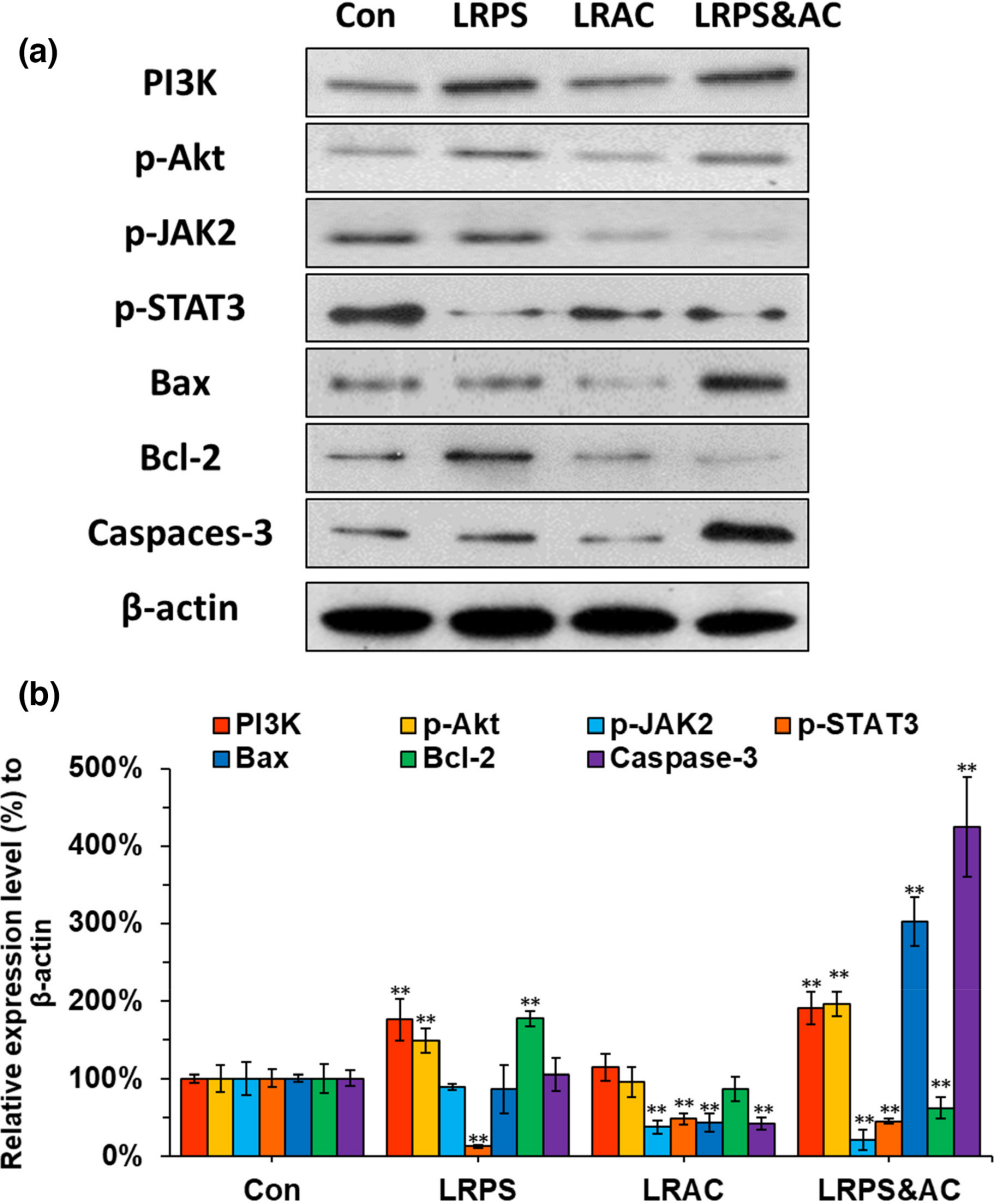
Expression levels of proteins involved in PI3K/Akt (phosphatidylinositol 3‐kinase/protein kinase B) and JAK2/STAT3 (janus kinase 2/signal transduction and activator of transcription 3) signaling pathways. (a) Western blotting results of PI3K, phosphorylated Akt (p‐Akt), phospho‐JAK2 (p‐JAK2), phospho‐STAT3 (p‐STAT3), BCL2 associated X protein (Bax), B‐cell lymphoma 2 (Bcl‐2), Caspase‐3, and β‐actin. (b) Relative expression levels of the proteins presented in (a). β‐Actin was used as the reference to calculate the relative gray values. The expression levels of the proteins in the control group were normalized to 100%. LRAC (20 μg/ml), LRPS (500 μg/ml), and LRPS&AC which was the mixture of LRAC (20 μg/ml) and LRPS (500 μg/ml). Asterisks denote a significant difference compared with that of the control group (*p < .05, **p < .01)

To dissect the molecular mechanism behind the antineoplastic effect of LRPS&AC, Western blotting was applied. Previous studies showed that polysaccharides from *Lycium barbarum* were able to act on PI3K/Akt signaling pathway, and JAK2/STAT3 in hepatoma cells could be triggered by anthocyanins (Baba et al., 2017; Yu et al., 2018). Therefore, proteins involving in the two pathways were monitored. As shown in Figure 7, when LoVo cells were treated with LRPS alone, the expression of PI3K and p‐Akt was significantly enhanced when comparing to that of the control (*p* < .01). Along with that, upregulation of Bcl‐2, the downstream effector of PI3K/Akt, was also observed. The expression of Caspase‐3, the hallmark for mitochondria‐related apoptosis, did not show significant difference relative to that of the control. This indicated that LRPS was not able to inhibit the LoVo cells, consistent with the MTT results of this study (Figure 3a). The cells treated with LRAC were observed with significant downregulations of p‐JAK2 and p‐STAT3 (*p* < .01), suggesting the activation of the signaling pathway by LRAC. However, no significant difference was detected in the level of Caspase‐3, which is probably due to the simultaneous downregulation of Bax and Bcl‐2 caused by LRAC. As a proapoptotic effector, Bax was able to induce cell apoptosis by enhancing the level of Caspase‐3. On the contrary, as encoded by an antiapoptotic gene, overexpression of Bcl‐2inhibits apoptosis in some carcinoma cells (Lo et al., 2007). When the cells were treated with LRPS&AC, proapoptotic Bax was significantly upregulated (*p* < .01), while the antiapoptotic Bcl‐2 was suppressed (*p* < .01), as a result of simultaneously initiated PI3K/Akt and JAK2/STAT3 pathways (Figure 7). The enhanced expression of Caspase‐3, as a downstream event of the elevated ratio of Bax/Bcl‐2, was observed (*p* < .01), resulting in the apoptosis of the LoVo cells as demonstrated in Figure 5. Besides, the apoptotic cells were also observed with the intracellular ROS accumulation, as shown in Figure 6. This was consistent with the previous report that ROS elevation and Caspase‐3 activation are closely related events (Luo et al., 2013). Polysaccharides from *L. barbarum* could downregulate the expression of Blc‐2 (Yang et al., 2019). In this study, the same phenomenon was observed in LoVo cells treated by LRPS. LRAC was observed with the ability to lower the level of Bax while leaving Bcl‐2 unaffected, which is similar to the effect of berry phenols on the HT‐29 cells (Wu et al., 2007). Taken together, the antineoplastic effects of LRPS&AC can be ascribed to the interaction of PI3K/Akt and JAK2/STAT3 pathways, ending up with increased the Bax/Bcl‐2 rate that dictates the mitochondria‐mediated apoptosis by boosting the level of Caspase‐3.

## 4 CONCLUSIONS

*Lycium ruthenicum* Murr. polysaccharides, polysaccharides extracted and purified from *L. ruthenicum*, were characterized by HPLC and FT‐IR. LRPS itself failed to exert antineoplastic activity on tumor cells, while synergistic antitumor effects were found when the LRPS was applied in combination with LRAC, *L. ruthenicum* anthocyanins. The mixture of LRPS and LRAC (LRPS&AC) could impede the progression of tumor cells (LoVo and HepG2) in a dose‐dependent fashion, without imposing any cytotoxicity on normal immune cells (RAW 264.7). Arresting cell cycle at the G0–G1 phase and inducing cell apoptosis via ROS‐dependent pathway were involved in the antitumor effects. The molecular mechanism behind the effects lies in the enhanced expression of Bax and downregulation of Bcl‐2, as a result of the crosstalk between PI3K/Akt and JAK2/STAT3 signaling pathways. This is the first study elucidating antineoplastic activities of natural products from *L. ruthenicum*, validating the healthcare and therapeutic efficacy of *L. ruthenicum*. Further in vivo studies using encapsulated LRPS&AC and the fruits of *L. ruthenicum* on mice are in progress.

## CONFLICT OF INTEREST

The authors declare that they have no conflict of interest.

## Supporting information

App S1

## Data Availability

The data that support the findings of this study are available on request from the corresponding author. The data are not publicly available due to privacy or ethical restrictions.
