# Supplementary material for: Synergistic antitumor effects of polysaccharides and anthocyanins from Lycium ruthenicum Murr. on human colorectal carcinoma LoVo cells and the molecular mechanism
Source: Food Sci Nutr. 2022 Apr 18;10(9):2956–68. doi: 10.1002/fsn3.2892 (PMC9469862; doi:10.1002/fsn3.2892)
Supplement: Supplementary file 1 — App S1 [file FSN3-10-2956-s001.docx]

**Supporting Information**

Synergistic antitumor effects of polysaccharides and anthocyanins from *Lycium ruthenicum* Murr. on human colorectal carcinoma LoVo cells and the molecular mechanism

Xinshu Qin^§^, Xingyu Wang^§,^*, Ke Xu^‡^, Xingbin Yang^§^, Qing Wang^†^, Chao Liu^†^, Xinkun Wang^†^, Xu Guo^†^, Jinyue Sun^†,^*, Lin Li^∫^, Shiqi Li^‖^

^†^ Key Laboratory of Novel Food Resources Processing, Ministry of Agriculture and Rural Affairs, Key Laboratory of Agro-Products Processing Technology of Shandong Province, Institute of Agro-Food Science and Technology, Shandong Academy of Agricultural Sciences, Ji’nan 250100, Shandong, China.

^§^ Shaanxi Engineering Laboratory for Food Green Processing and Safety Control, Shaanxi Key Laboratory for Hazard Factors Assessment in Processing and Storage of Agricultural Products, College of Food Engineering and Nutritional Science, Shaanxi Normal University, Xi’an 710062, Shaanxi, China.

^‡^ Department of Joint Surgery, Hong Hui Hospital, Xi’an Jiaotong University, Xi’an 710054, Shaanxi, China.

^∫^ Santa Barbara City College, University of California Santa Barbara, Santa Barbara 93106, California, USA.

^‖^ Department of Material Science and Engineering, Queen Mary University of London Engineering School, Northwestern Polytechnical University, Xi’an 710062, Shaanxi, China

* To whom correspondence should be addressed. Email: wangxingyu@snnu.edu.cn (X. Wang), moon_s731@hotmail.com (J. Sun);

**Content**

1. Extraction and purification of polysaccharides from *L. ruthenicum* Murr. …………………….............……………………..S2

2. Effect of LRPS with elevated concentration and prolonged incubation time on the proliferation of LoVo cells…………..S4

3. Characterization of anthocyanins from *L. ruthenicum* Murr. (LRAC) by HPLC ……….………...........…..................….…S5

4. LDH assay evaluating the permeability of LoVo cells membrane treated with LRPS&AC …..……….….………….….…S6

5. Antineoplastic effect of LRPS&AC on HepG2 cells …………….……......………………………...…...….…….……………S7

6. Antioxidant activity of LRPS against DPPH^•^, ABTS^•+^, OH^•^, and O_2_^•−^ ………………..…………................………………....S8

7. A campus questionnaire on *L. ruthenicum* Murr. and related functional foods ………………..…………………………....S10

8. Reference…………………………………………………………….……..…………….…………...…..….…..……………....S11

**1.** **Extraction and** **purification of polysaccharides from *L. ruthenicum* Murr.**

**Materials and Methods**

DEAE-Cellulose-52 was obtained from Solarbio Life Science Co. Ltd (Beijing, China). Sephadex G-75 medium was provided by Pharmacia Co. Ltd (Uppsala, Sweden). All the other reagents were purchased from Sinopharm Co. Ltd (Beijing, China), and used without further purification.

The polysaccharides from *L. ruthenicum* (LRPS) were prepared by using the following steps. First, *L. ruthenicum* powder of 150 g was dissolved in 500 mL SSC buffer (pH=3.0) containing 0.4% pectinase (w/v). The resulting mixture was placed in a shaking bath (100 r/min) at 65 °C for 2 h. After extraction, the solution was collected by vacuum filtration, and the residue was re-extracted two times using the same procedure. All the extraction solutions containing LRPS were combined and concentrated on a rotary evaporator at 45 °C for 1 h. Subsequently, ethanol (90%, v/v) of fourfold volume was slowly added to the concentrated solution to precipitate the polysaccharides. The polysaccharides sediment was re-dissolved and treated with Sevage reagent to remove proteins fractions, followed by dialyzed against distilled water for 48 h. The deproteinized solution was concentrated again and lyophilized under the vacuum conditions to obtain crude LRPS (CLRPS).

The CLRPS was then decolored according to the method with slight modifications ^[1]^. Certain amount of CLRPS was dissolved and kept in a shaking water bath (37 °C), followed by adjusting the pH to 8.8 using ammonia water. Then 30% of H_2_O_2_ was drop-wisely added until the color started to fade. The shaking continued for another 2 h, before solution turned into faint yellow. The resulting mixture was neutralized with 1.0 mol/L HCl, followed by dialysis against distilled water and lyophilization. The decolored CLRPS of 1.2 g was dissolved and loaded onto the anion-exchange column (DEAE-Cellulose-52, 5.0 cm×40 cm), followed by gradient elution of NaHCO_3_ at the flow rate of 1 mL/min. The concentration of NaHCO_3_ increased to 0.05 mol/L, 0.10 mol/L, 0.25 mol/L, and 0.50 mol/L, gradually. The eluted fractions were spectrophotometrically detected at 490 nm using the phenol-sulfuric acid method for polysaccharides, and at 280 nm using ultraviolet absorbance for protein. The distinct polysaccharides peaks were designated as LRPS1, LRPS2, LRPS3 and LRPS4, according to the chronological order. The main polysaccharides fraction, LRPS4 was subjected to next-step purification. LRPS4 of 60 mg was dissolved and loaded onto the Sephadex G-75 column (1.5 cm × 80 cm) using NaCl of 0.1 mol/L as eluent. The fractions were collected in tubes, and evaluated for the content of polysaccharides and protein, using phenol-sulfuric acid method and ultraviolet absorbance as described above, respectively. The fractions of the purified polysaccharides (LRPS4), as determined by phenol-sulfuric acid method, were pooled and concentrated. After lyophilization, LRPS4 of high purity was obtained.

**Results**

**
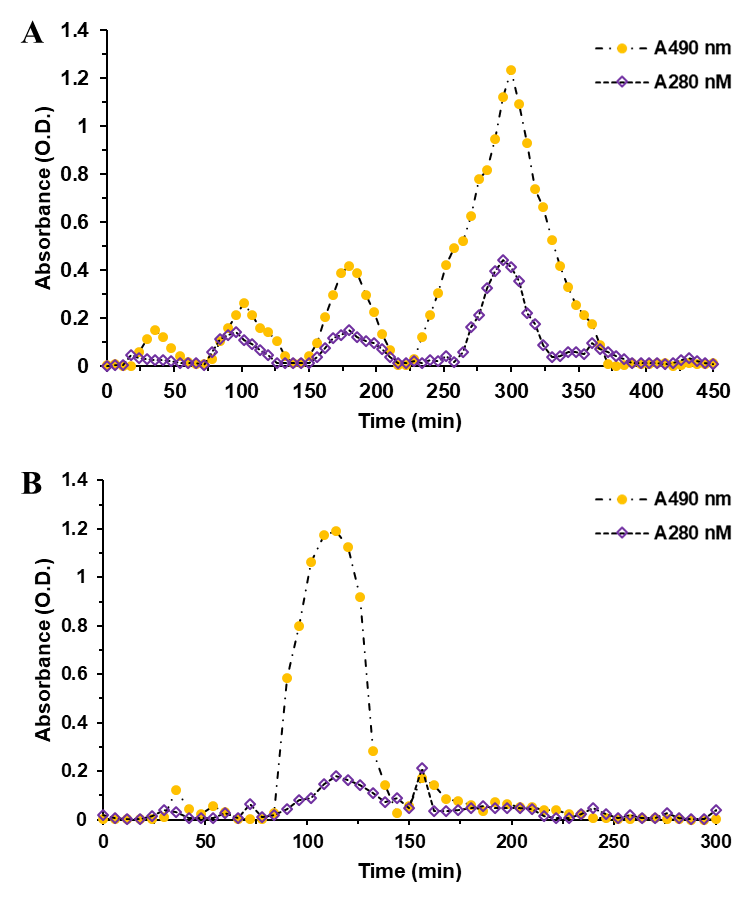
**

**Fig. S1. (A)** Elution profiles of LRPS loaded on DEAE-cellulose column by 280 nm and 490 nm. The four distinct peaks were designated as LRPS1, LRPS2, LRPS3, and LRPS4, respectively. **(B)** Elution profiles of LRPS4 using Sephadex G-75 column. Absorbance at 280 nm and 490 nm indicated the content of protein and polysaccharides, respectively.

**2.** **Effect of LRPS with elevated concentration and prolonged incubation time on the proliferation of LoVo cells**

**Materials and Methods**

The human colorectal carcinoma LoVo cells were incubated with varying concentration of LRPS (750 µg/mL, 900 µg/mL, 1200 µg/mL) at 37 ℃ in 5 % CO_2_ for 60 h and 72 h in RPMI-1640 medium, supplemented with 10% heat-inactivated FBS, 100 U/mL penicillin and 100 µg/mL streptomycin. The same volume of the medium instead of LRPS was added as the control group. Fluorouracil (5-FU) of 100 µg/mL was applied as the positive control. The MTT assay was used to measure viability of LoVo cells treated with LRPS. All the experiments on the LoVo cells were independently performed in triplicate.

**Results**

**
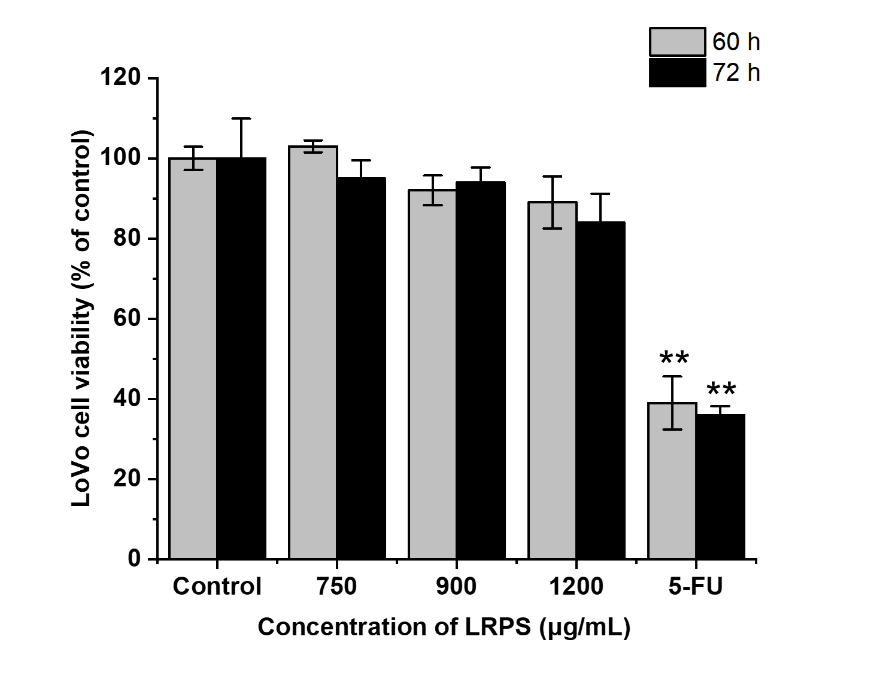
**

**Fig. S2. Cell viability of LoVo treated with different amount of LRPS for 60 h and 72 h**. Results were representative of three separated experiments, and were presented as means ± SD (n = 3). Asterisks denoted a statistical significance where **p*< 0.05, ***p*< 0.01 versus control.

**3.** **Characterization of anthocyanins from *L. ruthenicum* Murr. (LRAC) by HPLC**

**Materials and Methods**

The Zorbax SB-C18 column was purchased from Agilent Technologies (California, USA). All the reagents serving as mobile phases were chromatographically pure, provided by Thermo Fisher Scientific (Shanghai, China) and used without further treatment.

The purified LRAC was analyzed by Agilent 1200 HPLC system (Agilent Technologies, California, USA), equipped with Zorbax SB-C18 column (150 mm × 4.6 mm, 5 μM particle size). LRAC of 20 μL was injected, followed by measurement at 530 nm. Two mobile phases, A: 10% aqueous formic acid with 0.1% TFA, B: acetonitrile solution containing 15% methanol, were applied. The elution program was carried out as the following: 0-20 min, linear gradient of B from 5% to 12%; 20-30 min, linear gradient of B from 12% to 25%; 30-40 min, linear gradient of B from 25% to 60%; 40-60 min, linear gradient of B from 60% to 15%. The flow rate was set as 0.8 mL/min for the whole program, and the temperature of column chamber was kept at 30 °C invariably.

**Results**

**
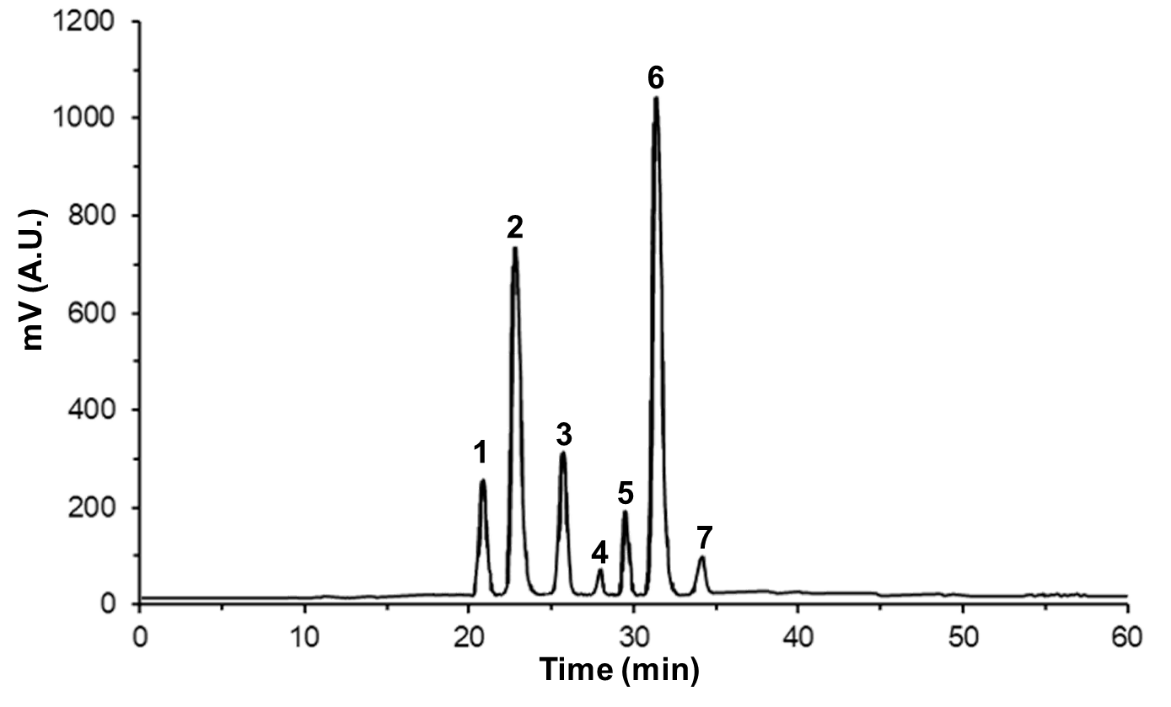
**

**Fig. S3.** Elution profile of LRAC analyzed by HPLC chromatograms at 525 nm. The distinct peaks were sequentially designated as one to seven according to their retention time.

**4.** **LDH assay evaluating the permeability of LoVo cells membrane treated with LRPS&AC**

**Materials and Methods**

The LRPS&AC with different concentrations (150 µg/mL, 300 µg/mL, and 500 µg/mL) were incubated with LoVo cells at 37 ℃ in 5% CO_2_ for 24 h and 48 h in RPMI-1640 medium. The same volume of the medium instead of LRAC was added to serve as control. The LDH assay was used to measure viability of LoVo cells after the treatment with LRAC, using the kit purchased from Nanjing JianCheng Bioengineering Co. Ltd. (Nanjing, China). All the experiments on the LoVo cells were performed in triplicate independently.

**Results**

**
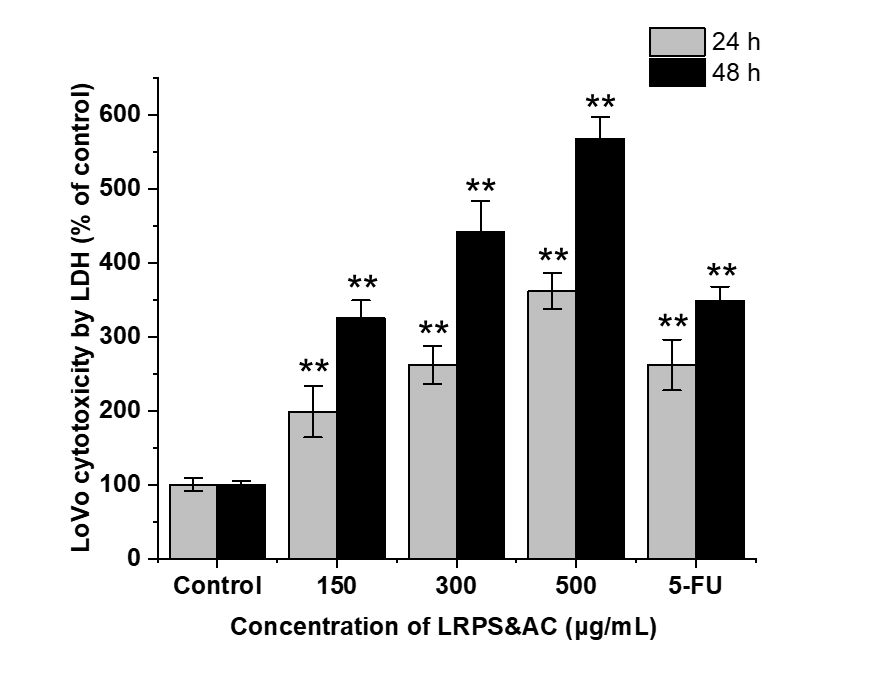
**

**Fig. S4. Cytotoxicity of LRPS&AC on LoVo cells measured by LDH leakage**. The LDH assay was applied to measure the viability of LoVo cells incubated with LRPS&AC. Results were representative of three separated experiments, and were presented as means ± SD (n = 3). Asterisks denoted a statistical significance where **p*< 0.05, ***p*< 0.01 versus control.

**5.** **Antineoplastic effect of LRPS&AC on HepG2 cells**

**Materials and Methods**

HepG2, a human hepatoma cell line, was purchased from Cell Bank of Institute of Biochemistry and Cell Biology, Chinese Academy of Sciences (Shanghai, China). The culture of the cell was based on the previous report with slight modification ^[2]^. The cells were revived in RPMI supplemented with 10% heat inactivated FCS, 2 g/L NaHCO_3_, 100 U/mL penicillin and 100 mg/L streptomycin. The cells were incubated at 37 ℃ in 5% CO_2_ to 90% confluence, followed by trypsin treatment with PBS solution containing 0.25% trypsin and 0.02% EDTA, and resuspended in the medium. The dissociative HepG2 cells were counted and 3×10^5^ cells in 100 µL of medium were seeded in a well on the 96-well microplate. When cells were grown to 70% confluence, serial concentrations of LRPS (150 µg/mL - 500 µg/mL) containing LRAC (20 µg/mL) were added. 5-FU (100 µg/mL) was used as positive control. The resulting mixtures were incubated for 24 h or 48 h, followed by cell viability test by MTT assay. All the experiments on the LoVo cells were performed in triplicate independently.

**Results**

**
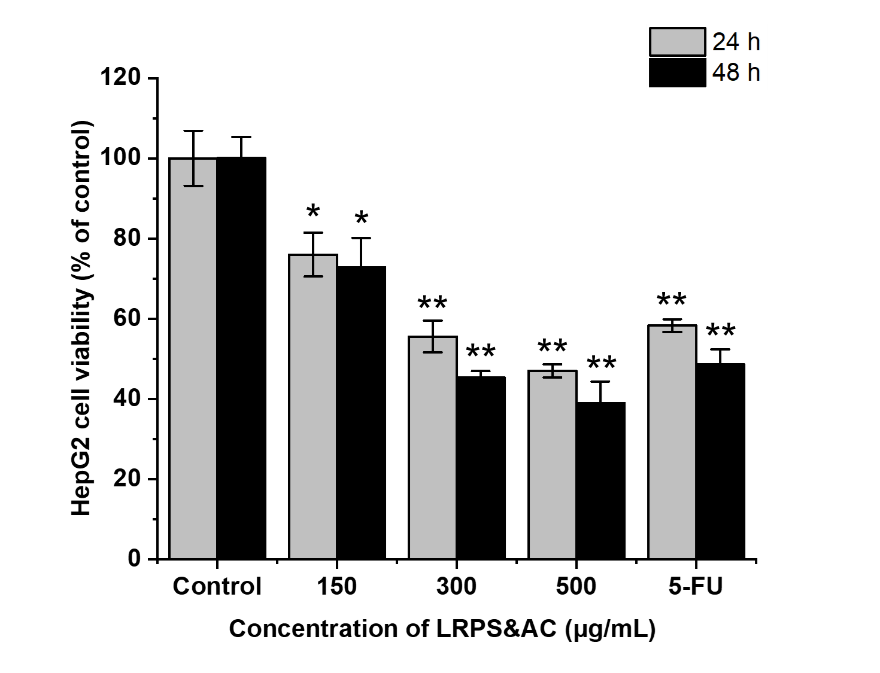
**

**Fig. S5. Cell viability of HepG2 treated with LRPS&AC varying in concentration**. The MTT assay was carried out to determine the cell viability of HepG2 incubated with different amount of LRPS&AC. Results were representative of three separated experiments, and were presented as means ± SD (*n* = 3). Asterisks denoted a statistical significance where **p*< 0.05, ***p*< 0.01 versus control.

**6.** **Antioxidant activity of LRPS against DPPH^•^, ABTS^•+^, OH^•^, and O_2_^•−^**

**Materials and Methods**

Trichloroaceticacid (TCA) and 1,1-diphenyl-2-picrylhydrazyl (DPPH) were purchased from Sigma (GmbH, Sternheim, Germany). Nitroblue tetrazolium (NBT), nicotinamideadenine dinucleotide (NADH) and phenazine methosulfate (PMS) were from Applichem (Darmstadt, Germany). EDTA was the products of Sinopharm Chemical Reagent Co. Ltd. (Shanghai, China). 2,2'-Azinobis-(3-ethylbenzthiazoline-6-sulphonate) (ABTS) was obtained from Merck (Darmstadt, Germany). All the other chemicals were of the highest grade available, and used without further purification.

**DPPH^•^ scavenging activity**

DPPH^•^ scavenging of LRPS was determined by the reported method ^[3]^. LRPS (80-800 µg/mL) of 2 mL was mixed with 2 mL DPPH^•^ methanol solution, and the resulting mixture was kept in dark for 30 min before subjecting to absorbance measurement at 517 nm. The scavenging ability was calculated as follows: scavenging activity against DPPH^•^ (%) = [1 - (A_1_ - A_2_)/A_0_] ×100%, where A_0_ was the absorbance of the control (LRPS replaced by equivoluminal deionized water), A_1_ was the absorbance in the presence of LRPS sample, A_2_ was the absorbance without DPPH^•^. Same volume of Vc (200 µg/mL) was used as a positive control. IC_50_, defined as LRPS concentration necessary to scavenge 50% of DPPH^•^, was obtained by plotting scavenging activity against LRPS concentration. Same process was applied to determine the IC_50_ values for the other three free radicals.

**ABTS^•+^ scavenging activity**

Briefly, ABTS*^•^*^+^ solution was prepared by mixing 10 mL of ABTS (7 mM) with 178 mL of K_2_S_2_O_8_ (140 mM), followed by being kept in dark overnight. One milliliter of LRPS samples of different concentrations was added to 2 mL of ABTS*^•^*^+^ to react in dark at room temperature for 10 min, then the absorbance at 732 nm was recorded. Vc was used instead of LRPS to serve as the positive control. The scavenging activity was formulated as following: ABTS*^•^*^+^ scavenging activity (%) = [1 - A_1_ /A_0_] × 100%, where A_0_ indicates absorbance of control (1.0 mL of distilled water instead of LRPS), A_1_ represents absorbance in the presence of LRPS sample.

**Superoxide anion radical scavenging activity**

Scavenging potential of LRPS on superoxide anion radical (O_2_^•−^) was measured according to the previous procedure ^[4]^. In brief, 1.0 mL of NBT, NADH and LRPS of different concentration were mixed, followed by addition of 0.4 ml of PMS. The resulting mixture was incubated at room temperature for 5 min, and the absorbance at 560 nm was measured. Scavenging activity of O_2_^•−^ was calculated according to the following equation: scavenging activity against O_2_^•−^ (%) = [1 - (A_1_ - A_2_)/A_0_] × 100%, where A_0_ indicates absorbance of control (1.0 mL of distilled water instead of LRPS), A_1_ represents absorbance in the presence of LRPS sample, A_2_ was absorbance without PMS. Same volume of Vc (200 µg/mL) instead of LRPS served as the positive control to validate the assay.

**Hydroxyl free radicals-scavenging activity**

The scavenging activity of LRPS on the hydroxyl radical (OH^•^) was measured by the protocol with some modifications ^[4]^. The reaction was performed in 10 mM PBS (pH 7.4), containing 2.8 mM deoxyribose, 2.8 mM H_2_O_2_, 25 µM FeCl_3_, 100 µM EDTA, and LRPS varying in concentration. Certain volume of ascorbic acid was added to the final concentration of 100 µM, followed by incubating the mixture at 37 °C for 1 h. After that, 1.0 mL of 1% thiobarbituric acid (TBA) and 1.0 mL of ice-cold 3.0% TCA were added. The resulting mixture was then placed in water bath at 95 °C for 20 min, followed by measuring the absorbance at 532 nm against D-Mannitol (designated as blank). The reaction without LRPS sample was used as negative control, therefore, OH•-scavenging activity (%) = [1 - A_1_ /A_0_] × 100%, where A_0_ was the absorbance of the negative control, A_1_ was the absorbance in the presence of LRPS. Same volume of Vc (200 g/mL) was used as the positive control.

**Results**

**
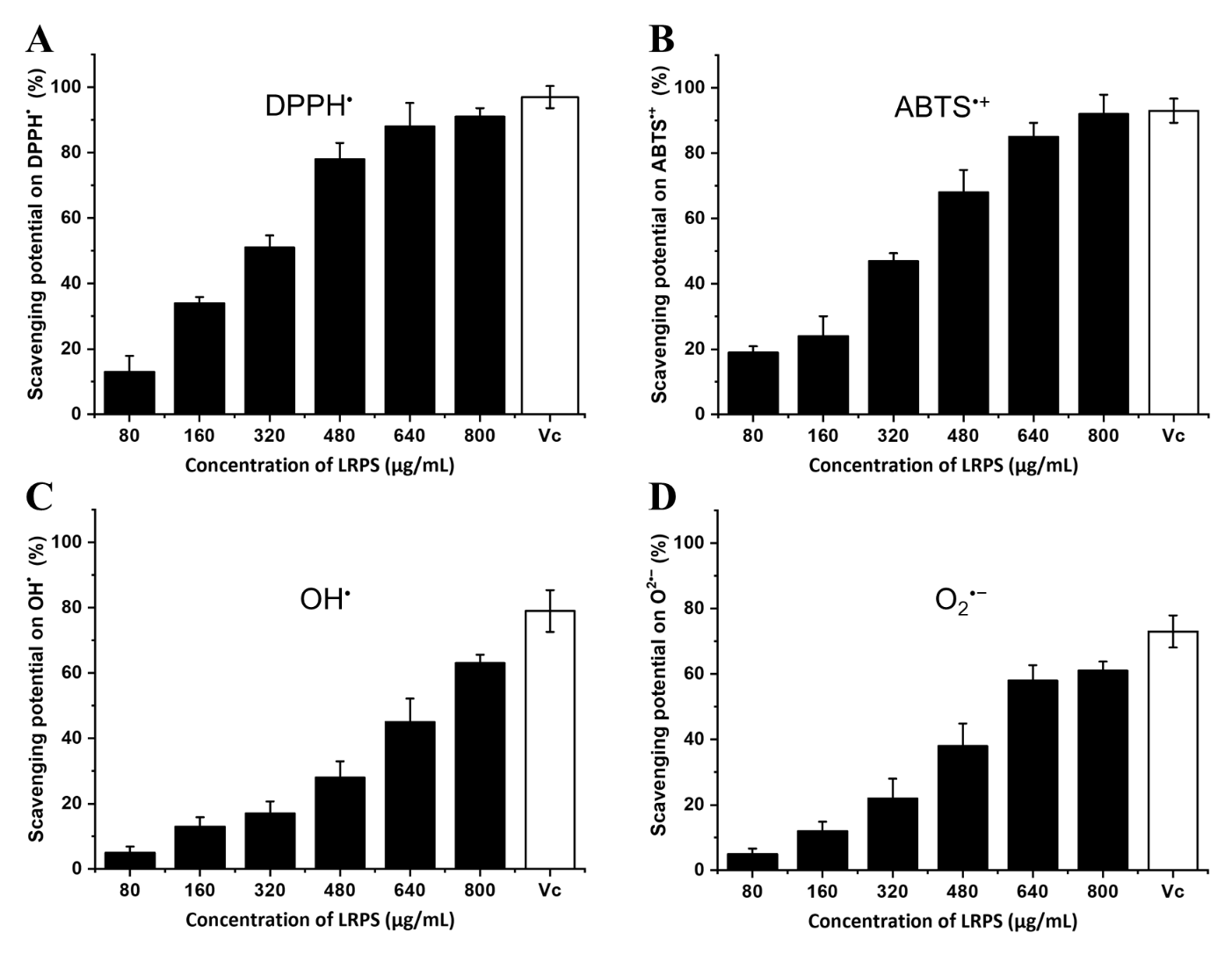
**

**Fig. S6.** Antioxidant activities of LRPS on DPPH^•^ **(A)**, ABTS^•+^ **(B)**, OH^•^ **(C)**, and O_2_^•−^ **(D)**. Vc was used as positive control. Results were representatives of three separated experiments and were presented as means ± SD (*n* = 3).

**7.** **A campus** **questionnaire on *L. ruthenicum* Murr. and related functional foods**

**Materials and Methods**

A questionnaire on the acceptance of goji fruits and related functional foods was designed and distributed in a campus. The survey was carried out among people at the age between 18 to 30 years old. More than 300 questionnaires were handed out among which 169 were regained. Screened by content analysis, 127 sheets were considered significant.

**Results**

**Table S1.** Statistical result of the survey on *L. ruthenicum* Murr. -based functional foods

| **Question** | **Positive** | **Negative** | **Not Matter** |
| --- | --- | --- | --- |
| 1. Are you familiar with Goji fruit? | 110 (86.3%) | 15 (11.8%) | 2 (1.9%) |
| 2. From your perspective, is Goji fruit a health-care food? | 116 (91.7%) | 6 (4.6%) | 5 (3.7%) |
| 3. Do you prefer black fruit Goji (*L. ruthenicum*) to red fruit Goji (*L. barbarum*) in terms of higher nutrients content? | 79 (62.1%) | 29 (23.2%) | 19 (14.7%) |
| 4. Are you willing to try health-care food derived from black fruit Goji (*L. ruthenicum*)? | 66 (51.8%) | 40 (31.8%) | 21 (16.4%) |
| 5. If the health promotion effect of the black fruit Goji-based functional food is significant, would you take it as a long-term nutritional enhancement? | 56 (44.3%) | 47 (36.8%) | 24 (18.9%) |

**Reference**

1. Peng Q, Lv X, Xu Q, *et al*. Isolation and structural characterization of the polysaccharide LRGP1 from *Lycium ruthenicum* [J]. *Carbohyd. Polym.*, **2012**, 90(1): 95-101.

2. Zhang M, Tang X, Wang F, Zhang Q, Zhang Z. Characterization of *Lycium barbarum polysaccharide* and its effect on human hepatoma cells [J]. *Int. J. Biol. Macromol.*, **2013**, 61, 270-275.

3. Tian L, Zhao Y, Guo C, *et al*. A comparative study on the antioxidant activities of an acidic polysaccharide and various solvent extracts derived from herbal *Houttuynia cordata* [J]. *Carbohyd. Polym.*,, **2011**, 83(2): 537-544.

4. Wang X, Gao A, Jiao Y, *et al*. Antitumor effect and molecular mechanism of antioxidant polysaccharides from *Salvia miltiorrhiza* Bunge in human colorectal carcinoma LoVo cells [J]. *Int. J. Biol. Macromol*, **2018**, 108: 625-634.
